# Seasonal and socio-demographic patterns of self-reporting major disease groups in north-west Burkina Faso: an analysis of the Nouna Health and Demographic Surveillance System (HDSS) data

**DOI:** 10.1186/s12889-021-11076-1

**Published:** 2021-06-09

**Authors:** Tobias Gottlieb-Stroh, Aurélia Souares, Till Bärnighausen, Ali Sié, Somkeita Pascal Zabre, Ina Danquah

**Affiliations:** 1 Institute for Social Medicine, Epidemiology and Health Economics, Charité – Universitätsmedizin Berlin, Berlin, Germany; 2 Heidelberg Institute of Global Health, Heidelberg University Hospital, Heidelberg, Germany; 3 Centre de Recherche en Santé de Nouna (CRSN), Nouna, Burkina Faso

**Keywords:** Double burden of disease, Sub-saharan Africa, Rural, Non-communicable diseases, Communicable diseases, Infectious diseases, Socio-demographic factors

## Abstract

**Background:**

Sub-Saharan Africa (SSA) is facing a rapid growth of non-communicable diseases (NCDs), while communicable diseases still prevail. For rural SSA, evidence for this development is scarce. We aimed at quantifying self-reported major disease groups according to season, and determining the associations with socio-economic factors in rural Burkina Faso.

**Methods:**

This study used data of 4192 adults (age range: 18–101 years; male: 49.0%) from the Nouna Health and Demographic Surveillance System (HDSS) in north-west Burkina Faso, rainy season of 2010 and dry season of 2011. We assessed the proportions and their 95% confidence intervals (CIs) of self-reported major disease groups as defined by the World Health Organization. For their associations with socio-economic factors, odds ratios (OR), 95% CIs and *p*-values were calculated by logistic regression.

**Results:**

The surveys were completed by 3949 adults in 2010 (mean age: 37.5 ± 14.9 years, male: 48.8%) and by 4039 adults in 2011 (mean age: 37.3 ± 16.2 years, male: 49.1%). The proportions of self-reported communicable diseases were 20.7% (95% CI: 19.4–21.9%) in the rainy season and 11.0% (10.0–11.9%; McNemar’s *p* < 0.0001) in the dry season. Self-reported NCDs amounted to 5.3% (4.6–6.0%) and 4.5% (3.8–5.1%; *p* = 0.08), respectively. In each year, less than 1% reported injuries (*p* = 0.57). Few individuals reported an overlap of communicable diseases and NCDs: 1.4% in 2010 and 0.6% in 2011. In the multiple-adjusted models, formal education (vs. lack of education) showed the strongest association with self-reporting of communicable diseases in both seasons. For NCD-reporting, non-manual occupation (vs. manual) was positively associated, only in the rainy season.

**Conclusions:**

Self-reporting of communicable diseases is subject to seasonal variation in this population in north-west Burkina Faso. The exact reasons for the low overall self-reporting of NCDs and injuries, apart from a low socio-demographic position, require further investigation.

## Introduction

Historically, the predominant health problems in sub-Saharan Africa (SSA) have been infectious, maternal, perinatal and nutritional conditions, henceforth defined as communicable diseases [1]. For some decades, however, the region has experienced an upsurge of metabolic diseases, cancers, mental illnesses and other non-communicable diseases (NCDs) [2]. Indeed, in 2004, more than half of the deaths in SSA were caused by communicable diseases and one quarter by NCDs [3]. In 2017, the burden of NCDs according to disability-adjusted life-years (DALYs) was almost equivalent to that of communicable diseases [4]. In fact, NCDs are estimated to exceed communicable diseases as the most common cause of death in this geographic region by 2030 [3]. However, data from rural sub-Saharan Africa describing this Double Burden of Disease are lacking.

Clearly, SSA health systems have to turn towards this emerging phenomenon [5]. This is challenging, because the local systems have developed in response to acute illnesses, and most of the already under-resourced budget is spent on the fight against communicable diseases. Training and expertise of health personnel is equally focused on communicable diseases, and building-up capacity to cope with NCDs has been neglected for long [6]. In addition, better understanding about the interactions between communicable diseases and NCDs in SSA [7] is warranted to equip policy makers with empirical evidence on where to invest the country’s limited resources for health care.

In the absence of objective and extensive prevalence measures, self-reported diagnoses constitute inclusive sources of information for capturing clinical and subclinical conditions [8, 9]. They reflect more than just the pure absence of disease. In fact, the auto-declaration of illnesses by questioned individuals is one of the only methods available for evaluating the morbidity of populations in resource-poor settings [10, 11].

Furthermore, disease occurrence in SSA may depend on seasonal variation and thus, impact public healthcare efforts. For communicable diseases, this phenomenon is well described for common diseases such as malaria and tuberculosis [12, 13]. Due to the chronic nature of NCDs, their occurrence and self-report should not vary by season. Yet, the severity of symptoms may differ according to climatic and harvest seasons, leading to changes in self-reporting of the respective conditions. This might relate to the worsening of cardio-vascular conditions during heat waves [14], and the development of vaso-occlusive crisis in sickle cell patients during cold weather [15]. Also, self-reported injuries may be prone to seasonal variation, especially in rural SSA [16].

So far, there is little evidence for the perceived and real occurrence of major disease groups in rural areas and non-hospital settings in SSA [17]. Therefore, we aimed at identifying the occurrence and co-occurrence of major disease groups in two different seasons, and the associations with socio-demographic factors among adults living in rural north-west Burkina Faso, mainly for the creation and prioritization of public health policies to be put in place.

## Methods

### Study design

The Nouna Health and Demographic Surveillance System (HDSS) has been established in 1992 for systematic collection of longitudinal data of the population living in the Nouna Health District. This HDSS comprises 59 contiguous villages over 1775 km^2^ with a representative population of around 11,373 households. In regular intervals, the Nouna HDSS assesses natality, mortality, as well as in- and out-migration.

### Study area and population

This study used data of the Nouna HDSS in north-western Burkina Faso. As of 2007, the population comprises around 115,000 inhabitants of different ethnicities and religious beliefs, most of which depend on subsistence farming. Almost one-third of the population lives in the semi-urban town Nouna, the only location where grid electricity and piped water exist. The area is characterized by a sub-Sahelian climate with one dry season (November to May) and one rainy season (June to October). In 2010, there were 13 primary health facilities in HDSS area, and one district hospital. The mean distance to the nearest health center is about 8.5 km, translating into a 75-min-walk in the dry season and a 90-min-walk in the rainy season [18, 19].

### Sampling and data collection

The study was conducted between June and October 2010, and between November and May 2011. We used a two-stage sampling in the rural areas. The first stage selected villages, called primary units, from the list of villages. Following this, the second stage selected the households to survey, based on the list of households in each primary unit. This type of sampling was chosen rather than a simple random due to feasibility for travel between villages. Indeed, two-stage sampling allows for the number of surveyed villages to be reduced and so to limit the geographical disparity of sample households, which, from a logistics point of view, simplifies the organisation and the sensitization of heads of households in preparation for the survey and the movements of the interviewers. It is true that two-stage sampling is less precise due to the ‘cluster effect’ if the groups are not homogenous (high inter-group variability). Meanwhile, this loss of precision has been anticipated and compensated for by the increase in the size of the sample. Moreover, in the studied area, the socio-cultural characteristics of the households are overall homogenous [20]. Since 2003, a household survey, covering basic socio-economic and basic health data among others has been conducted among a random sample of 10% of the HDSS households (*n* = 1400) during the dry season for each year. Only for the present study, and to allow for comparisons between climatic seasons, demographic and socio-economic data as well as information about acute and chronic diseases were recorded during the rainy season of 2010 and the dry season of 2011. More specifically, amongst the total number of households which took part in the HDSS census (11,373), 7743 were distributed over 58 villages, i.e. 68% in rural households and 3630 lived in the city of Nouna, i.e. 32% semi-urban. As a consequence, to insure the representation in the sample of rural and semi-urban households from the census, 674 rural households and 316 semi-urban households were taken as samples with a total of 990 household (8.7% of HDSS population). To ensure representativeness of the sample, we replaced households that moved, emigrated or split after the rainy season in 2010 by another randomly chosen household of the same village and the same size.

Households were visited by trained field staff. For questionnaire-based interviews, we have used an inventory of chronic and acute illnesses declared by each member of the households. For each illness of the past 4 weeks, several characteristics were collected, such as symptoms, duration, care, degree of severity, direct and indirect costs, to measure the weight of illnesses on the health of the household. We included all adults above the age of 18 years, who completed the acute and chronic disease modules.

### Statistical analysis

We analysed the two cross-sectional surveys for all adults who participated in the questionnaire-based interviews either in 2010 or in 2011. The general characteristics of the study population are presented as categorical information and in percentages (95% confidence intervals, CIs). The data are presented per each season, for the total population and separately for men and women. Self-reported diseases were categorized into NCDs, communicable diseases and injuries according to the WHO major disease groups [1]. For the rainy season in 2010 and for the dry season in 2011, the disease proportions and their 95% CIs were calculated for major disease groups and for the three most frequent diseases per group, respectively. To assess the differences of major disease groups between the seasons we used McNemar’s test. Differences between men and women were assessed by χ^2^-test. In addition, we calculated the overlaps between self-reported major disease groups for the rainy season and the dry season. For each season and each of the WHO major disease groups, namely communicable diseases, NCDs and injuries, we fitted logistic regression models to determine crude associations (Model 1) with demographic and socio-economic factors. Based on these findings and scientific evidence [21], we controlled for potential confounders in multiple-adjusted models (Model 2). The analyses were adjusted for study location (i.e. village code) to account for the cluster design. As a sensitivity analysis, we additionally adjusted for household identification code to rule out the effect of shared households.

## Results

### Study population

Table 1 presents the general characteristics of the study population. In 2010, 3949 adults completed the survey; this figure was 4039 participants in 2011. The mean age was 37.5 ± 14.9 years in 2010 and 37.3 ± 16.2 years in 2011. There were 49% men in both seasons. Also, 58% of all adults were not able to read, and 76% have a lack of formal education.

**Table 1:** General characteristics of adults in the Nouna Health and Demographic Surveillance System 2010 and 2011

Characteristics	2010 (Rainy Season)			2011 (Dry Season)		
	Total	Male	Female	Total	Male	Female
N	3949	48.8 (47.0–50.0)	51.2 (50.0–53.0)	4039	49.1 (48.0–51.0)	50.9 (49.0–52.0)
**Age group (years)**						
18–28	38.5 (37.0–39.9)	39.5 (37.3–41.7)	37.5 (35.4–39.6)	38.9 (37.4–40.4)	39.8 (37.7–42.0)	37.9 (35.8–40.0)
29–38	23.1 (21.8–24.4)	24.1 (22.2–26.0)	22.1 (20.3–23.9)	23.1 (21.8–24.4)	24.3 (22.4–26.2)	21.9 (20.1–23.7)
39–48	14.4 (13.3–15.5)	14.5 (12.9–16.0)	14.4 (12.9–15.9)	14.6 (13.5–15.7)	14.3 (12.8–15.9)	14.8 (13.3–16.4)
49–58	10.5 (9.5–11.4)	9.5 (8.2–10.8)	11.4 (10.0–12.8)	10.5 (9.6–11.5)	9.4 (8.1–10.7)	11.6 (10.2–13.0)
59–68	7.4 (6.6–8.2)	7.1 (5.9–8.2)	7.7 (6.6–8.9)	7.0 (6.2–7.8)	6.8 (5.7–7.9)	7.3 (6.2–8.4)
> 68	6.2 (5.4–6.9)	5.4 (4.4–6.5)	6.8 (5.7–7.9)	5.9 (5.2–6.7)	5.4 (4.4–6.4)	6.5 (5.4–7.5)
**Residence**						
Village	68.1 (66.7–69.6)	68.3 (66.2–70.4)	68.0 (66.0–70.0)	68.0 (66.6–69.5)	68.3 (66.3–70.4)	67.7 (65.7–69.8)
Nouna town	31.9 (30.4–33.3)	31.7 (29.6–33.8)	32.0 (30.0–34.0)	32.0 (30.6–33.4)	31.7 (29.6–33.7)	32.3 (30.3–34.3)
**Ethnic group**						
Dafin	32.2 (30.8–33.7)	33.0 (30.9–35.1)	31.5 (29.5–33.6)	33.1 (31.7–34.6)	34.1 (32.0–36.2)	32.2 (30.2–34.2)
Bwaba	29.1 (27.7–30.6)	29.3 (27.3–31.3)	29.0 (27.0–31.0)	28.9 (27.5–30.3)	29.1 (27.1–31.1)	28.7 (26.8–30.7)
Mossi	19.9 (18.7–21.2)	18.8 (17.1–20.6)	20.9 (19.2–22.7)	19.4 (18.2–20.6)	18.3 (16.6–20.0)	20.4 (18.7–22.1)
Samo	8.2 (7.3–9.0)	8.6 (7.3–9.8)	7.8 (6.6–8.9)	8.4 (7.5–9.2)	8.7 (7.5–10.0)	8.0 (6.9–8.2)
Peulh	7.4 (6.6–8.2)	7.6 (6.4–8.8)	7.3 (6.1–8.4)	7.2 (6.4–8.0)	7.1 (6.0–8.2)	7.3 (6.1–8.4)
Other	3.1 (2.6–3.7)	2.7 (2.0–3.5)	3.5 (2.7–4.3)	3.0 (2.5–3.6)	2.7 (2.0–3.4)	3.4 (2.6–4.2)
**Religion**						
Muslim	55.5 (53.9–57.0)	55.0 (52.8–57.3)	55.9 (53.7–58.0)	56.2 (54.7–57.7)	55.5 (53.3–57.7)	56.8 (54.7–59.0)
Catholic	30.0 (28.6–31.3)	29.5 (27.5–31.6)	30.4 (28.4–32.4)	29.3 (27.9–30.7)	29.1 (27.1–31.1)	29.5 (27.5–31.5)
Protestant	7.3 (6.5–8.1)	7.2 (6.0–8.3)	7.5 (6.4–8.7)	7.3 (6.5–8.1)	7.1 (5.9–8.2)	7.5 (6.4–8.7)
Animistic	6.7 (6.0–7.5)	7.8 (6.6–9.0)	5.7 (4.7–6.8)	6.8 (6.0–7.6)	7.9 (6.7–9.0)	5.7 (4.7–6.8)
Other	0.5 (0.2–0.7)	0.5, 0.2–0.8)	0.4 (0.2–0.7)	0.4 (0.2–0.7)	0.5 (0.2–0.8)	0.4 (0.1–0.7)
**Ability to read**						
Yes	26.9 (25.5–28.3)	39.6 (37.4–41.8)	14.7 (13.2–16.2)	26.8 (25.5–28.2)	39.2 (37.0–41.3)	14.8 (13.3–16.4)
No	58.3 (56.7–59.8)	46.7 (44.5–49.0)	69.3 (67.3–71.3)	58.3 (56.8–59.9)	46.8 (44.6–49.0)	69.5 (67.5–71.5)
Unknown	14.9 (13.7–16.0)	13.6 (12.1–15.2)	16.0 (14.4–17.6)	14.9 (13.8–16.0)	14.0 (12.5–15.5)	15.7 (14.1–17.3)
**Educational level**						
None	75.5 (74.1–76.8)	73.0 (71.0–75.0)	77.8 (76.0–79.7)	75.7 (74.4–77.0)	73.1 (71.2–75.1)	78.2 (76.5–80.0)
Primary School	9.5 (8.6–10.4)	12.3 (10.9–13.8)	6.7 (5.6–7.8)	9.4 (8.5–10.3)	12.0 (10.6–13.5)	6.8 (5.7–7.9)
Secondary School	0.6 (0.3–0.8)	1.0 (0.5–1.4)	0.2 (0.0–0.5)	0.5 (0.3–0.8)	0.9 (0.5–1.3)	0.2 (0.0–0.4)
Tertiary School	0.1 (0.0–0.2)	0.2 (0–0.4)	0 (0)	0.1 (0–0.2)	0.2 (0.0–0.3)	0 (0)
Unknown	14.4 (13.3–15.5)	13.5 (12.0–15.0)	15.2 (13.6–16.8)	14.3 (13.2–15.4)	13.8 (12.3–15.3)	14.8 (13.2–16.3)
**Occupation**						
Agriculture	64.0 (62.5–65.5)	70.7 (68.7–72.7)	57.7 (55.5–59.9)	64.3 (62.8–65.8)	70.6 (68.6–72.6)	58.2 (56.0–60.3)
Other manual labor	3.1 (2.6–3.7)	3.7 (2.8–4.5)	2.6 (1.9–3.3)	3.2 (2.7–3.8)	3.8 (3.0–4.7)	2.6 (1.9–3.3)
Non-manual labor	7.7 (6.9–8.6)	4.5 (3.5–5.4)	10.8 (9.5–12.2)	7.7 (6.9–8.6)	4.4 (3.5–5.3)	11.0 (9.6–12.3)
Other/ unknown	25.1 (23.8–26.5)	21.2 (19.3–23.0)	28.9 (26.9–30.9)	24.8 (23.4–26.1)	21.2 (19.4–23.0)	28.2 (26.3–30.2)

Data are presented as percentage (95% confidence intervals)

### Proportion and co-occurrence of self-reported diseases according to season

Table 2 shows the proportions of self-reported disease groups and the three most common diseases in each group. Communicable diseases were reported among 21% (95% CI: 19–22%) of the participants in the rainy season. This figure was almost halved in the dry season: 11% (95% CI: 10–12%; McNemar’s *p* < 0.0001). Malaria was the most common self-reported communicable disease, followed by cold and dermatophytosis. For self-reported NCDs, 5.3% (95% CI: 4.6–6.0%) of the participants were affected in the rainy season compared to 4.5% (95% CI: 3.8–5.1%) in the dry season (*p =* 0.08). Hypertension predominated in both years, followed by chronic heart diseases and rheumathoid arthritis. Injuries were reported by 0.8% (95% CI: 0.5–1.0%) of the survey participants in 2010 and by 0.6% (95% CI: 0.4–0.9%) in 2011 (*p* = 0.57). As for differences between men and women, only NCD-reporting in 2010 (rainy season) was significantly more frequent among women (*p* < 0.001). This was mainly attributed to chronic heart diseases (*p* = 0.002).

**Table 2:** Proportions of self-reported diseases among adults in the Nouna Health and Demographic Surveillance System 2010 and 2011

**2010 (Rainy Season)**	**Total (3949)**	**Male (1928)**	**Female (2021)**	**χ**^**2**^ ***p*-value**
**Non-communicable diseases**	5.3 (4.6–6.0)	4.0 (3.1–4.9)	6.5 (5.5–7.6)	<.0001
Hypertension	0.9 (0.6–1.2)	0.7 (0.4–1.1)	1.1 (0.7–1.6)	0.179
Chronic Heart Disease	0.8 (0.6–1.1)	0.4 (0.1–0.7)	1.3 (0.8–1.8)	0.002
Rheumathoid Arthritis	0.5 (0.3–0.7)	0.5 (0.2–0.8)	0.6 (0.3–0.9)	0.583
Others (< 20 cases)	2.9 (2.4–3.5)	2.4 (1.7–3.1)	3.5 (2.7–4.3)	0.045
**Communicable diseases**	20.7 (19.4–21.9)	20.8 (19.0–22.6)	20.5 (18.8–22.3)	0.837
Malaria	15.7 (14.6–16.8)	17.1 (15.4–18.8)	16.2 (14.6–17.8)	0.455
Cold	1.2 (0.9–1.5)	1.3 (0.8–1.8)	1.1 (0.7–1.6)	0.554
Dermatophytosis	0.6 (0.4–0.8)	0.5 (0.2–0.8)	0.7 (0.4–1.1)	0.376
Others (< 20 cases)	2.4 (1.9–2.8)	2.0 (1.4–2.6)	2.7 (2.0–3.4)	0.120
**Injuries**	0.8 (0.5–1.0)	0.8 (0.4–1.2)	0.7 (0.3–1.1)	0.620
Paralysis	0.2 (0.0–0.3)	0.2 (0.0–0.4)	0.1 (0.0–0.2)	0.382
Snake bite	0.1 (0.0–0.2)	0.2 (0.0–0.3)	0.0 (0.0–0.0)	0.076
Trauma by accident	0.1 (0.0–0.3)	0.2 (0.0–0.3)	0.2 (0.0–0.3)	0.954
Others (< 20 cases)	0.4 (0.2–0.6)	0.3 (0.1–0.6)	0.5 (0.2–0.7)	0.493
**2011 (Dry Season)**	**Total (4039)**	**Male (1985)**	**Female (2054)**	**χ**^**2**^ ***p*-value**
**Non-communicable diseases**	4.5 (3.8–5.1)	3.9 (3.1–4.8)	5.0 (4.0–5.9)	0.111
Hypertension	0.7 (0.4–0.9)	0.5 (0.2–0.8)	0.9 (0.5–1.3)	0.099
Chronic Heart Disease	0.5 (0.3–0.8)	0.4 (0.1–0.7)	0.7 (0.3–1.0)	0.229
Rheumathoid Arthritis	0.4 (0.2–0.6)	0.3 (0.0–0.5)	0.6 (0.3–0.9)	0.103
Others (< 20 cases)	2.8 (2.3–3.3)	2.8 (2.1–3.6)	2.8 (2.1–3.5)	0.996
**Communicable diseases**	11.0 (10.0–11.9)	10.6 (10.0–12.7)	11.3 (10.0–12.7)	0.437
Malaria	5.8 (5.1–6.6)	6.1 (5.0–7.2)	5.6 (4.6–6.6)	0.501
Cold	2.5 (2.0–3.0)	2.8 (2.1–3.6)	2.2 (1.6–2.8)	0.200
Dermatophytosis	0.6 (0.4–0.9)	0.4 (0.1–0.7)	0.9 (0.5–1.3)	0.060
Others (< 20 cases)	2.0 (1.6–2.5)	1.3 (0.8–1.8)	2.8 (2.1–3.5)	0.001
**Injuries**	0.6 (0.4–0.9)	0.6 (0.3–1.0)	0.6 (0.3–1.0)	0.908
Paralysis	0.3 (0.1–0.4)	0.2 (0.0–0.3)	0.3 (0.1–0.6)	0.225
Snake bite	0.1 (0.0–0.2)	0.1 (0.0–0.2)	0.2 (0.0–0.3)	0.682
Trauma by accident	0.1 (0.0–0.2)	0.2 (0.0–0.3)	0.0 (0.0–0.0)	0.078
Others (< 20 cases)	0.2 (0.0–0.3)	0.2 (0.0–0.4)	0.2 (0.0–0.3)	0.672

Data are presented as percentage (95% confidence interval). Comparisons between males and females were made by χ^2^-test

Diseases with < 20 cases were summarized as “other” (Table 2). In 2010, 18.9% of participants reported diseases that could not be classified into any of the WHO major disease groups, because the participants did not know the kind of illness (715/743), reported to have had “a symptom affecting the whole body” (12/743), to had been bewitched (2/743), or other reasons (14/743). In 2011, there were 23.0% of participants with self-reported diseases without classification. The reasons were unknown illness (938/943) and others (5/943).

**Fig. 1:**
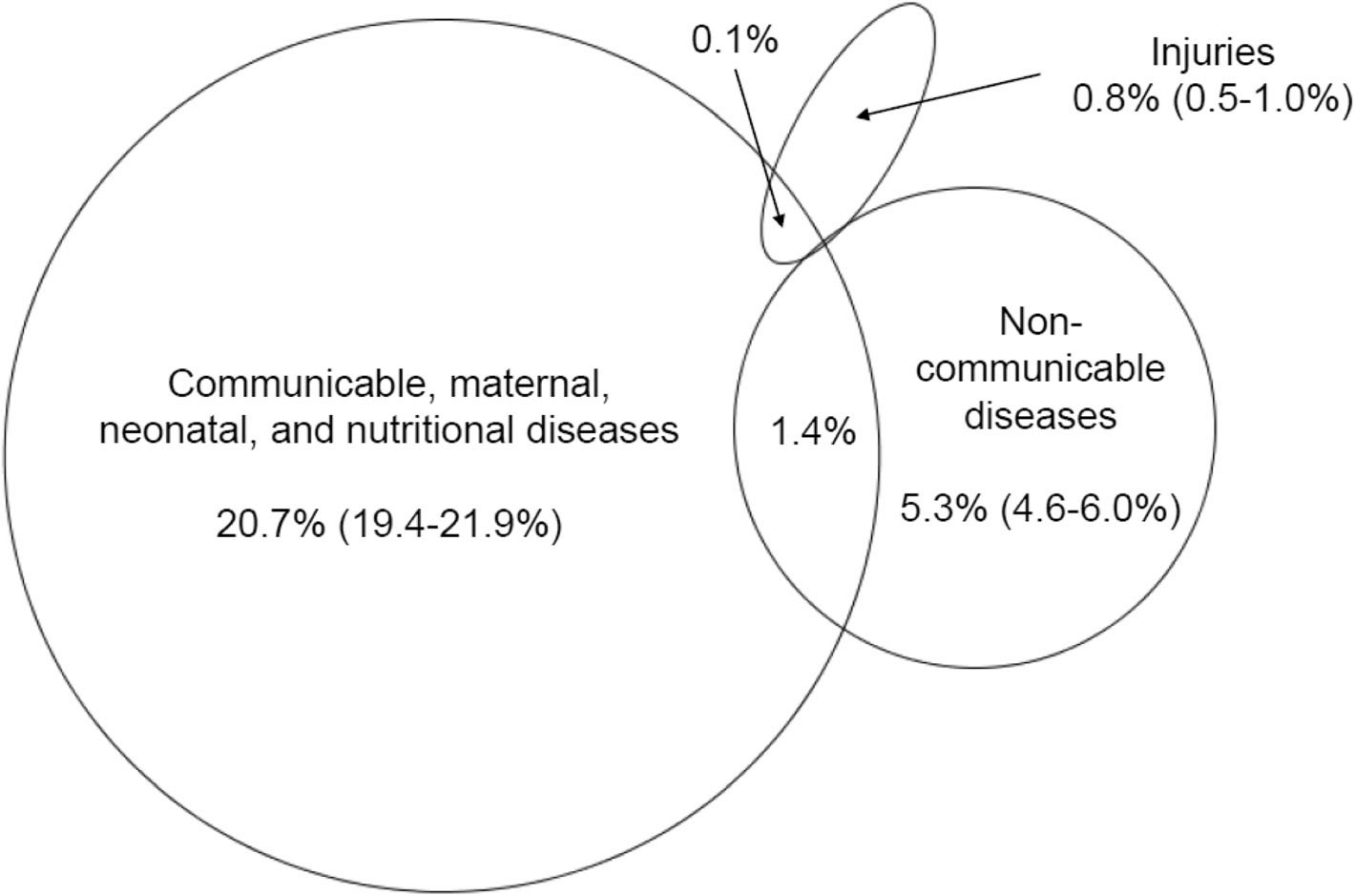
Co-occurrence of self-reported disease groups in the Nouna HDSS. rainy season of 2010

**Fig. 2:**
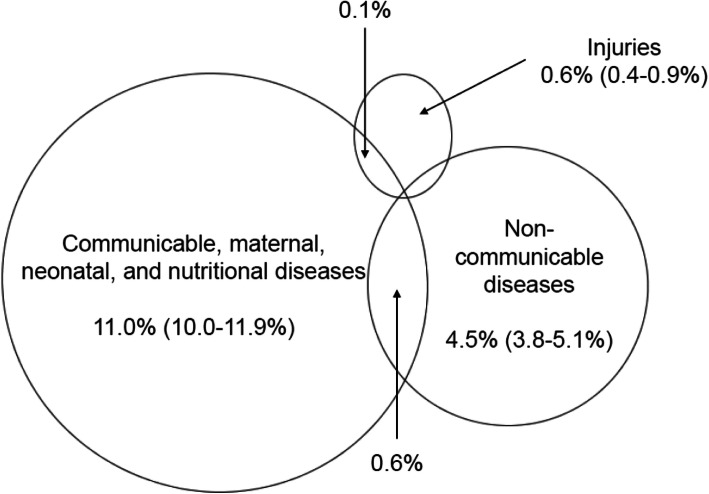
Co-occurrence of self-reported disease groups in the Nouna HDSS. dry season of 2011

The Venn diagrams in Figs. 1 and 2 display the co-occurrences of self-reported major disease groups during the rainy season and during the dry season. In the rainy season, 1.4% of adults reported to have had a communicable disease and an NCD at the same time, while this number amounted to 0.6% for the dry season. The overlaps between self-reported communicable diseases and injury-reporting were each 0.1%. There were no overlaps between NCD- and injury-reporting. The most common co-occurrence of self-reported diseases was malaria and hypertension in both years.

### Associations with demographic and socio-economic factors

**Fig. 3:**
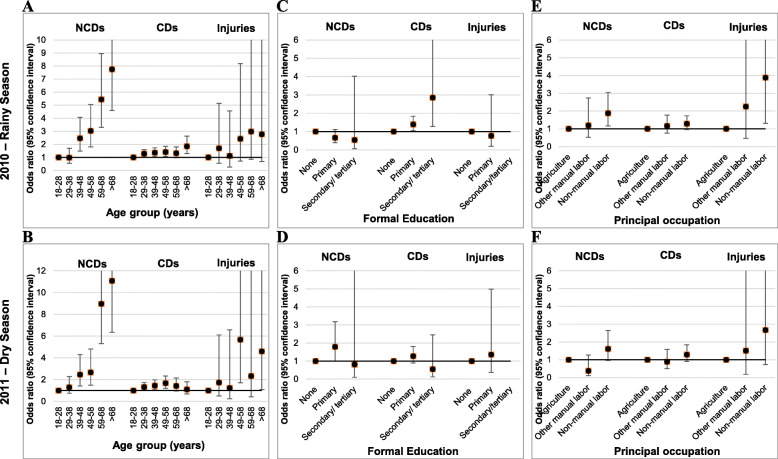
Multiple-adjusted associations with self-reported disease groups among adults of the Nouna HDSS

Table 3 shows the univariate associations of demographic and socio-economic factors with self-reported NCDs for the rainy season and the dry season. In both seasons, we observed the highest odds of self-reported NCDs with older age, followed by non-manual occupation (vs. agriculture), and residence in Nouna town (vs. village). These associations attenuated in the multivariate models (Fig. 3A-B, E-F). Females showed higher odds of self-reported NCDs than men, but this was only seen in the rainy season (OR: 1.68; 95% CI: 1.26–2.24). The associations of ethnic group with NCD self-report were neither consistent between univariate and multivariate models nor between the rainy and the dry seasons (Table 3).

**Table 3:** Univariate associations with self-reported non-communicable diseases in the Nouna HDSS in 2010 (rainy season) and 2011 (dry season)

Factor	2010 (3949)						2011 (4039)					
	% NCDs	% in non-NCD	% in NCD	OR	95% CI	***p***-value	% NCDs	% in non-NCD	% in NCD	OR	95% CI	***p***-value
**Age group (years)**												
18–28	2.2	39.7	16.3	1.00			1.8	39.9	16.1	1.00		
29–38	2.4	23.8	10.5	1.08	0.63–1.86	0.779	2.5	23.5	12.8	1.35	0.77–2.34	0.293
39–48	6.0	14.4	16.3	2.77	1.70–4.50	<.0001	4.4	14.6	14.4	2.45	1.43–4.20	0.001
49–58	7.7	10.2	15.3	3.66	2.23–6.00	<.0001	4.9	10.5	11.7	2.76	1.56–4.89	0.001
59–68	14.0	6.7	19.6	7.13	4.44–11.46	<.0001	14.8	6.3	23.3	9.22	5.64–15.09	<.0001
> 68	18.9	5.3	22.0	10.2	6.39–16.28	<.0001	16.3	5.2	21.7	10.31	6.24–17.04	<.0001
**Sex**												
Male	4.0	49.5	36.8	1.00			3.9	49.4	43.3	1.00		
Female	6.5	50.5	63.2	1.68	1.26–2.24	<.0001	5.0	50.6	56.7	1.28	0.94–1.73	0.111
**Residence**												
Village	4.8	68.5	61.2	1.00			4.0	68.3	61.1	1.00		
Nouna town	6.4	31.5	38.8	1.38	1.04–1.84	0.028	5.4	31.7	38.9	1.37	1.01–1.87	0.043
**Ethnic group**												
Dafin	4.2	32.6	25.4	1.00			4.2	33.2	31.1	1.00		
Bwaba	5.5	29.1	30.1	1.33	0.92–1.94	0.132	3.9	29.1	25.0	0.92	0.61–1.37	0.676
Mossi	5.1	19.9	19.1	1.23	0.81–1.88	0.327	4.0	19.5	17.2	0.94	0.60–1.48	0.800
Samo	8.4	7.9	12.9	2.11	1.30–3.41	0.002	6.8	8.2	12.8	1.67	1.01–2.76	0.044
Peulh	5.1	7.4	7.2	1.24	0.69–2.24	0.470	5.2	7.1	8.3	1.25	0.70–2.24	0.456
other	10.3	3.0	5.3	2.24	1.14–4.41	0.020	9.4	2.9	5.6	2.03	1.01–4.08	0.048
**Religion**												
Muslim	4.7	55.8	49.8	1.00			4.6	56.1	57.8	1.00		
Catholic	6.2	29.7	34.9	1.32	0.97–1.79	0.079	4.5	29.3	29.4	0.98	0.70–1.37	0.890
Protestant	5.5	7.3	7.7	1.17	0.68–2.01	0.567	2.7	7.4	4.4	0.58	0.28–1.20	0.144
Animistic	5.3	6.7	6.7	1.11	0.63–1.98	0.711	4.7	6.8	7.2	1.04	0.57–1.87	0.904
**Formal education**												
None	6.4	74.6	90.9	1.00			5.0	75.3	85.6	1.00		
Primary	4.3	9.6	7.7	0.66	0.39–1.11	0.114	5.0	9.3	10.6	0.99	0.61–1.62	0.985
Secondary/ tertiary	3.6	0.7	0.5	0.54	0.07–4.02	0.544	4.0	0.6	0.6	0.79	0.11–5.85	0.814
**Principal occupation**												
Agriculture	4.5	64.5	55.0	1.00			4.0	64.6	58.3	1.00		
Other manual labor	5.7	3.1	3.3	1.27	0.58–2.78	0.555	2.3	3.3	1.7	0.56	0.17–1.79	0.328
Non-manual labor	10.2	7.3	14.8	2.37	1.57–3.60	<.0001	9.3	7.4	16.1	2.42	1.58–3.72	<.0001
Other/ unknown	5.6	25.0	26.8	1.26	0.91–1.74	0.174	4.3	24.8	23.9	1.07	0.74–1.53	0.730

Odds ratios, 95% confidence intervals and *p*-values were calculated by logistic regression

In Table 4, we present the univariate associations for self-reported communicable diseases. We observed a positive gradient between older age groups and self-reported communicable diseases. These were stronger in the rainy season than in the dry season. The OR for self-reported communicable diseases was 1.91 (95% CI: 1.01–1.40) among individuals living in Nouna town as compared to participants living in rural areas. But this was only seen for the univariate model in the rainy season. Also, in the rainy season, primary education (vs. none) was positively associated with self-reported communicable diseases in the univariate (OR: 1.28; 95% CI: 1.01–1.63) as well as the multivariate models (OR: 1.39; 95% CI: 1.01–1.87). This association was not seen in the dry season (Fig. 3C-D). In the univariate models, non-manual labor (vs. agriculture) was directly associated with self-reported communicable diseases in the rainy season (OR: 1.57; 95% CI: 1.21–2.03) and in the dry season (OR: 1.45; 95% CI: 1.05–2.00). However, this was not seen in the multivariate models (Fig. 3E-F).

**Table 4:** Univariate associations with self-reported communicable diseases in the Nouna HDSS in 2010 (rainy season) and 2011 (dry season)

Factor	2010 (3949)						2011 (4039)					
	% CDs	% in non-CD	% in CD	OR	95% CI	***p***-value	% CDs	% in non-CD	% in CD	OR	95% CI	***p***-value
**Age group (years)**												
18–28	16.5	40.5	30.6	1.00			8.5	39.9	30.7	1.00		
29–38	22.1	22.7	24.6	1.44	1.17–1.77	0.001	11.7	22.8	25.1	1.43	1.09–1.86	0.008
39–48	23.2	14.0	16.2	1.53	1.21–1.94	<.0001	12.4	14.3	16.9	1.54	1.14–2.07	0.005
49–58	24.2	10.0	12.3	1.62	1.24–2.10	<.0001	14.4	10.1	14.0	1.80	1.31–2.48	<.0001
59–68	24.0	7.1	8.6	1.60	1.18–2.16	0.002	12.7	6.9	8.4	1.58	1.07–2.33	0.021
> 68	25.9	5.7	7.7	1.78	1.29–2.44	<.0001	9.2	6.1	5.0	1.06	0.66–1.71	0.797
**Sex**												
Male	20.8	48.7	49.1	1.00			10.3	49.4	47.4	1.00		
Female	20.5	51.3	50.9	0.98	0.84–1.15	0.84	11.1	50.6	52.6	0.92	0.76–1.13	0.437
**Residence**												
Village	19.7	68.9	65.1	1.00			10.2	68.4	65.0	1.00		
Nouna town	22.7	31.1	34.9	1.19	1.01–1.40	0.035	11.8	31.6	35.0	1.16	0.95–1.43	0.152
**Ethnic group**												
Dafin	24.2	30.8	37.7	1.00			11.4	32.9	35.2	1.00		
Bwaba	20.3	29.3	28.7	0.80	0.66–0.97	0.023	11.1	28.8	30.0	0.97	0.76–1.25	0.837
Mossi	14.4	21.5	13.8	0.53	0.41–0.67	<.0001	8.7	19.8	15.8	0.74	0.55–1.00	0.051
Samo	18.9	8.3	7.5	0.73	0.54–0.99	0.047	9.5	8.5	7.4	0.82	0.55–1.22	0.325
Peulh	22.5	7.2	8.1	0.91	0.67–1.23	0.546	11.4	7.1	7.7	1.01	0.68–1.49	0.975
other	31.8	2.9	4.2	1.18	0.78–1.79	0.426	15.1	2.9	3.8	1.21	0.71–2.08	0.478
**Religion**												
Muslim	21.1	55.2	56.5	1.00			10.1	56.6	53.0	1.00		
Catholic	21.3	29.7	30.9	1.01	0.85–1.20	0.874	11.8	28.9	32.3	1.19	0.95–1.48	0.123
Protestant	18.3	7.6	6.5	0.84	0.61–1.15	0.274	12.5	7.1	8.6	1.28	0.89–1.85	0.187
Animistic	18.0	7.0	5.9	0.83	0.59–1.15	0.254	8.8	6.9	5.6	0.87	0.56–1.34	0.525
**Formal education**												
None	23.3	72.9	85.2	1.00			11.7	74.9	82.4	1.00		
Primary	28.1	8.6	12.9	1.28	1.01–1.63	0.042	14.0	9.0	12.2	1.23	0.90–1.67	0.195
Secondary/ tertiary	46.4	0.5	1.6	2.85	1.35–6.02	0.006	8.0	0.6	0.5	0.64	0.15–2.73	0.548
**Principal occupation**												
Agriculture	22.4	62.7	69.4	1.00			11.6	63.7	69.3	1.00		
Other manual labor	27.6	2.8	4.2	1.32	0.88–1.99	0.174	11.5	3.2	3.4	0.97	0.56–1.69	0.921
Non-manual labor	31.1	6.7	11.6	1.57	1.21–2.03	0.010	16.0	7.3	11.5	1.45	1.05–2.00	0.024
Other/ unknown	12.2	27.8	14.8	0.48	0.39–0.60	<.0001	6.8	25.9	15.8	0.66	0.43–0.74	<.0001

Odds ratios. 95% confidence intervals and *p*-values were calculated by logistic regression

Finally, we show the univariate associations for self-reported injuries in Table 5. In the rainy season, the reporting of injuries was more likely among individuals with advanced age (> 68 years vs. 18–28 years) in both, the univariate (OR: 4.22; 95% CI: 1.18–15.06) and the multivariate models (Fig. 3A). This was also seen in the dry season (OR: 5.30; 95% CI: 1.41–19.90 and Fig. 3D). The associations between religion and injury-reporting were not consistent across seasons and changed upon adjustment in the multivariate models (Fig. 3E-F).

**Table 5:** Univariate associations with self-reported injuries in the Nouna HDSS in 2010 (rainy season) and 2011 (dry season)

Factor	2010 (3.949 adults)						2011 (4.039 adults)					
	% injuries	% in non-injuries	% in injuries	OR	95% CI	***p***-value	% injuries	% in non-injuries	% in injuries	OR	95% CI	***p***-value
**Age group (years)**												
18–28	0.4	38.6	20.0	1.00			0.3	39.0	20.0	1.00		
29–38	0.8	23.1	23.3	1.95	0.65–5.83	0.230	0.5	23.1	20.0	1.69	0.49–5.85	0.408
39–48	0.5	14.5	10.0	1.33	0.33–5.35	0.684	0.3	14.6	8.0	1.07	0.21–5.51	0.939
49–58	1.2	10.4	16.7	3.08	0.94–10.15	0.064	1.6	10.4	28.0	5.24	1.65–16.60	0.005
59–68	1.7	7.3	16.7	4.39	1.33–14.49	0.015	0.7	7.0	8.0	2.22	0.43–11.50	0.342
> 68	1.6	6.1	13.3	4.22	1.18–15.06	0.027	1.7	5.9	16.0	5.30	1.41–19.90	0.013
**Sex**												
Male	0.8	48.8	53.3	1.00			0.6	49.2	48.0	1.00		
Female	0.7	51.2	46.7	0.83	0.41–1.71	0.620	0.6	50.8	52.0	1.05	0.48–2.30	0.908
**Residence**												
Village	0.7	68.2	63.3	1.00			0.5	68.1	52.0	1.00		
Nouna town	0.9	31.8	36.7	1.24	0.59–2.61	0.571	0.9	31.9	48.0	1.97	0.90–4.33	0.091
**Ethnic group**												
Dafin	0.6	32.3	23.3	1.00			0.5	33.2	28.0	1.00		
Bwaba	1.0	29.1	36.7	1.74	0.67–4.52	0.251	0.8	28.8	36.0	1.48	0.55–3.98	0.440
Mossi	1.0	19.9	26.7	1.86	0.67–5.15	0.232	0.6	19.4	20.0	1.22	0.39–3.86	0.733
Samo	0.6	8.2	6.7	1.13	0.23–5.47	0.879	0.6	8.4	8.0	1.13	0.23–5.47	0.878
Peulh	0.3	7.5	3.3	0.62	0.08–5.05	0.655	0.7	7.2	8.0	1.32	0.27–6.39	0.730
other	0.9	3.1	3.3	1.47	0.18–12.05	0.719	0.0	3.1	0	–	–	–
**Religion**												
Muslim	0.7	55.5	50.0	1.00			0.5	56.3	44.0	1.00		
Catholic	0.9	29.9	36.7	1.36	0.62–2.97	0.441	1.1	29.1	52.0	2.28	1.02–5.12	0.045
Protestant	0.0	7.4	0	–	–	–	0.0	7.3	–	–	–	–
Animistic	1.5	6.7	13.3	2.21	0.73–6.72	0.161	0.4	6.8	4.0	0.75	0.10–5.85	0.785
**Formal education**												
None	0.9	75.4	90.0	1.00			0.6	75.7	76.0	1.00		
Primary	0.8	9.5	10.0	0.88	0.27–2.93	0.841	1.1	9.3	16.0	1.71	0.58–5.04	0.334
Secondary/ tertiary	0.0	0.7	0	0	–	–	0.0	0.6	–	–	–	–
**Principal occupation**												
Agriculture	0.7	64.1	56.7	1.00			0.5	64.4	48.0	1.00		
Other manual labor	1.6	3.1	6.7	2.44	0.56–10.69	0.236	0.8	3.2	4.0	1.67	0.21–12.94	0.624
Non-manual labor	2.0	7.6	20.0	2.96	1.16–7.58	0.023	1.3	7.7	16.0	2.79	0.89–8.70	0.077
Other/ unknown	0.5	25.2	16.7	0.75	0.27–2.03	0.570	0.8	24.7	32.0	1.74	0.71–4.26	0.228

Odds ratios. 95% confidence intervals and *p*-values were calculated by logistic regression

In a sensitivity analysis, we additionally adjusted for the household identification code in our final regression models to account for potential effects of shared households. The strength and the direction of the effect estimates marginally changed.

## Discussion

In this study, we assessed the occurrence and co-occurrence of WHO major disease groups, based on self-reported diseases and their associations with socio-demographic factors among 4000 adults in rural Burkina Faso. Between 2010 and 2011, we found that self-reported communicable diseases were frequent (11–21%) and subject to seasonal variation, while NCD- and injury-reporting were only seen in a small fraction of the study population (5 and 1%, respectively).

For communicable diseases, our findings reflect the Annual Statistical Report of the Ministry of Health of Burkina Faso [22]. The ten most frequent reasons for consultation in basic health facilities are mainly due to communicable diseases, largely due to malaria (48%). For NCDs, however, our results contradict the phenomenon of the Double Burden of Disease and the fact that NCDs accounted for 33% of all deaths in this country in 2012 [3, 22].

There might be two possible explanations for this finding. One being that the low occurrence of self-reported NCDs reflect the actual low prevalence of such conditions in Nouna. This may indicate that the ‘Double Burden of Disease’ has not arrived to this region yet. Secondly, the actual prevalence of NCDs in the region is higher but has not been reported due to a lack of awareness among participants.

The socio-demographic characteristics displayed in Table 1 argue for an actual low prevalence of NCDs. Low levels of literacy, lack of formal education and high subsistence farming rates point towards more traditional lifestyles. In fact, only half of our population were able to read and write, and three-quarters of our participants had never received formal education. Therefore, NCD-risk factors like western diet and sedentary lifestyles might have not affected the Nouna region yet, and thus, have not caused the ‘Double Burden of Disease’. This is supported by the univariate associations for NCDs and people living in Nouna town as compared to those living in villages. Urbanization can lead to a more sedentary lifestyle and self-subsistence farming is replaced by occupations that are physically less demanding [23].

Alternatively, low literacy rates and a lack of formal education can also point towards a lack of awareness towards NCDs. Historically those conditions have not existed and therefore people might not be able to interpret symptoms accordingly. This has been seen in other rural communities in West-Africa: Only 7.4% of patients with hypertension in Ghana were aware of their condition [24]. Especially rural communities have been shown to have an even lower awareness compared to urban dwellers [25]. The same is true for health facilities. A lack of diagnostic means and experience with NCDs might lead to underdiagnosis.

Additionally, we found a strong direct association of educational attainment and occupational level with NCD-reporting in our study. This supports both arguments described above. The better off might be more prone to the behavioral risk factors of NCDs such as western diet reduced physical activity because they use car and motorbike, and thus, have a higher NCD-prevalence. On the other hand, higher educational levels might just as well give them an increased awareness towards new disease forms. Their NCD-reporting might therefore be more frequent compared to that of less educated counterparts.

The seasonal variation of self-reported communicable diseases, mainly malaria, might reflect the environmental conditions affecting the reproduction of *Anopheles spc.* This is well described in the literature [26]. It is also conceivable that some NCDs and injuries vary with season. For instance, cardio-vascular conditions have been shown to be associated with increasing temperatures [14]. Similarly, subsistence farming populations in rural West Africa are more frequently exposed to injuries related to agricultural activities, such as snakebites and work accidents [16]. However, such variations were not discernible in our study population.

Furthermore, we found that the most common combination of self-reported diseases was malaria and hypertension. Indeed, Burkina Faso remains a high-transmission country (≥1 case per 1000 population), as reported for 2011 [12]. However, the co-occurrence with hypertension leaves room for speculation about an etiological link. In fact, a study in Côte d’Ivoire has found an association of malaria symptom severity and blood pressure [27]. Etyang et al. give further possible explanations for this link: Malaria contributes to low birth-weight, malnutrition and chronic inflammation in early-life – all these factors are associated with hypertension [28].

In contrast to previous studies in West-Africa, we did not observe co-occurrence of other diseases. For instance, among Ghanaian patients with diabetes mellitus, 46% showed an increased risk of infection with *Plasmodium falciparum* as compared to participants who had no diabetes mellitus [29]. Also, we have not seen typical associations between diabetes and tuberculosis, between HIV and tuberculosis, and between diabetes mellitus and HIV [30–32]. Low awareness or lack of diagnostic means for such conditions could again explain these null findings. Additionally, stigmatization associated with specific disease diagnosis, such as HIV, could lead to underreporting.

For self-reported communicable diseases, we can only speculate about the association with the ethnicity of Mossi. Further investigations are needed in this regard. The association of communicable diseases with older age might stem from less efficiently working immune systems among older adults [33]. The higher odds for individuals living in Nouna Town can also only be speculated about and further investigation about a lack of preventive action is needed. However, overreporting due to better awareness is possible as well. The same is true for the association with higher education and non-manual labor. Both reflect higher socio-economic status, leading to increased disease awareness and possibly more frequent reporting.

Injury reporting was associated with older age which even though atypical can be explained by lower bone density and a higher risk of falling in the elderly [34, 35]. The association with non-manual labor has not been expected as manually working individuals should be expected to have more accidents [36].

### Strengths and limitations

This work is based on a large, representative morbidity survey among adults (*N* = 4192) living in the Nouna Health District [18]. While the study has already been conducted in 2010/2011, the available morbidity data constitute the most comprehensive information about the health status in this population. Still, our findings may forfeit their validity over time. All measures of disease occurrence were based on the self-reports of our participants. This means our estimates do not reflect prevalence. Self-reporting can lead to misclassification of health conditions such as the classification of any condition with fever as a malaria episode. It is also prone to different interpretations among different cultures and can cause recall bias as it depends on the memory of each individual. However, there is a value in self-reported disease occurrence. In low-income countries where resources are scarce, it can be the only option to obtain information. In the Nouna area, only 0.6% of the population consult a doctor for acute health issues, and health insurance data is lacking [37]. If health policies should address this region, self-reported data constitute the best available information source. Self-reporting also has the advantage of depicting the actual needs of participants and their healthcare uptake [38].

Furthermore, in cross-sectional analyses, temporal relationships cannot be assessed, and thus, this study cannot comment on causality. Similarly, we cannot exclude the possibility of chance findings in our univariate models, which assessed several risk factors. Yet, in the multiple-adjusted models, we included only the most important socio-economic risk factors. We have adjusted for well-established confounders but cannot rule-out unmeasured or residual confounding.

Therefore, further efforts are needed to investigate the disease burden of rural populations in SSA and self-reporting should be complemented with other diagnostic means.

## Conclusions

In this population in rural Burkina Faso, NCD-reporting was comparatively low, and further investigations are needed to confirm these findings. It is clear that efforts in the fight against communicable diseases need to be intensified, especially in rural areas surrounding Nouna town. Low NCD-reporting does not mean that the danger of a *Double burden of Disease* in these regions is unreal. Measures to prevent these conditions are needed either way, because prevention is always better than cure. This should also include raising awareness for NCDs and increasing health literacy, so that individuals and health facilities are able to respond to the health challenges ahead.

## Data Availability

The datasets used and/or analysed during the current study are available from the corresponding author on reasonable request.

## Abbreviations

CI: Confidence interval
CRSN: Centre de Recherche en Santé de Nouna
HDSS: Health and Demographic Surveillance System
HIGH: Heidelberg Institute of Global Health
NCD: Non-communicable disease
OR: Odds ratio
SSA: Sub-Saharan Africa
